# Human Papillomavirus Assays and Cytology in Primary Cervical Screening of Women Aged 30 Years and Above

**DOI:** 10.1371/journal.pone.0147326

**Published:** 2016-01-20

**Authors:** Matejka Rebolj, Jesper Bonde, Sarah Preisler, Ditte Ejegod, Carsten Rygaard, Elsebeth Lynge

**Affiliations:** 1 Department of Public Health, University of Copenhagen, Copenhagen, Denmark; 2 Department of Pathology, Copenhagen University Hospital, Hvidovre, Denmark; 3 Clinical Research Centre, Copenhagen University Hospital, Hvidovre, Denmark; Universidad Nacional de la Plata, ARGENTINA

## Abstract

In women aged ≥30 years, Human Papillomavirus testing will replace cytology for primary cervical screening. We compared Hybrid Capture 2 (HC2), cobas, CLART, and APTIMA HPV assays with cytology on 2869 SurePath samples from women undergoing routine screening at 30–65 years in Copenhagen, Denmark. Women with cytological abnormalities were managed according to routine recommendations, with 92% completeness. Those with cytology-normal/HPV-positive samples (on any of the four assays) were invited for repeated cytology and HPV testing in 1.5 year, and 58% had additional testing. HPV testing detected more ≥CIN3 than cytology (HC2: 35, cobas, CLART: 37, APTIMA: 34, cytology: 31), although statistically the differences were not significant. Cobas and CLART detected significantly more ≥CIN2 than cytology (cobas, CLART: 49, cytology: 39). The proportion of women with false-positive test results (positive test results without ≥CIN3) varied between 3.3% with cytology and 14.9% with cobas. All HPV assays led to significantly more false-positive tests, whereas compared to HC2 cobas and CLART were associated with a significantly higher and APTIMA with a significantly lower proportion. Detection of CIN1 was particularly increased for the three DNA assays. With APTIMA combined with cytological triage, about 20% more women were referred for colposcopy than with cytology screening. With the three DNA assays, the increase was ≥50%. The number of women with repeated testing was twice as high with APTIMA and almost five times as high with cobas compared to cytology. To our knowledge, Horizon was the only study set in routine practice that compared more than two HPV assays in the same women while also ascertaining the histological status of women with normal cytology/HPV-positive test results. HPV-based screening of Danish women aged 30–65 detected more high-grade CIN but decreased the screening specificity, and increased the demand for additional testing.

## Introduction

Detection of high-risk Human Papillomavirus (HPV) infections is a more sensitive cervical cancer screening method than the current standard of cytology [1]. However, most infections clear spontaneously without leading to high-grade cervical intraepithelial neoplasia (CIN) [2]. HPV infections are most common in young women [3–5] who have a relatively low immediate risk of cervical cancer. Therefore, women aged ≥30 years are considered to be of a particular interest for HPV-based screening, as they have both fewer HPV infections and a higher risk of cervical cancer. Many HPV assays are now commercially available [6], and need to be thoroughly evaluated and compared before they are implemented in primary screening.

The aim of our study was to compare four commercially available HPV assays with cytology in primary screening of women aged 30–65 years. We used data from the Danish Horizon study, which was nested into routine screening and compared Hybrid Capture 2 High-Risk HPV DNA Test (HC2; QIAGEN, Gaithersburg, MD), cobas HPV Test (Roche Diagnostics, Pleasanton, CA), CLART HPV2 Assay (Genomica, Madrid, Spain), APTIMA mRNA HPV Test (Hologic, San Diego, CA), and cytology. In this study, women with normal cytology and positive HPV test results had repeated testing. Unlike other major studies comparing several HPV assays in primary screening which only assessed histological outcomes in women with abnormal cytology [7], in the Horizon study women with normal cytology and positive HPV test results were managed with repeated testing.

## Materials and Methods

### Study design

The design of the Horizon study was described in detail previously [3–5,8,9]. In short, the target number of samples was set to 5000, based on capacity and processing considerations. In total, 5064 consecutive SurePath samples arriving for routine liquid-based cytology (LBC) analysis at the Department of Pathology of Copenhagen University Hospital, Hvidovre, in June-August 2011 were tested with all four HPV assays. The approximately 2 ml of residual original material were diluted with 2 ml of SurePath. A single sample was available from 5005 (99%) women, whereas 59 samples (1%) were from 29 women, for whom the most abnormal sample was used (having the most abnormal cytology, or the highest number of positive HPV test results out of four in case of normal cytology). Primary samples were defined, by linkage to the national Pathology Data Bank (Patobank) [10], as those without: a previous cervical cancer, CIN in ≤3 years, atypical squamous cells of undetermined significance (ASCUS) or non-CIN cervical biopsy in ≤15 months, or a more severe cytological abnormality, inadequate cytology or a positive HPV test in ≤12 months. Reflecting routine practice, primary samples were predominantly screening samples but included a small proportion of samples taken for investigation of symptoms.

Women were recommended for cervical screening every three years until age 49, and every five years until age 65, and those included in the Horizon study were managed according to cytology and HPV test results. Women with abnormal cytology were followed according to the routine Danish guidelines, which recommended repeated testing in case of low-grade squamous intraepithelial lesions (LSIL; these women were not routinely tested for HPV DNA) or HPV-negative ASCUS, or a colposcopy in case of other cytological abnormalities. At this laboratory, HC2 was routinely used for HPV triage of ASCUS. Per study protocol, women with normal cytology and a positive test result on at least one of the four HPV assays at baseline were invited for repeated cytology and HPV testing in approximately 1.5 years, in November 2012. Additional information was provided on woman’s request by phone or e-mail by one of the investigators (JB) or the laboratory’s medical staff overseeing the screening program in the Capital Region (including CR). A reminder for follow-up was sent in March 2013. At follow-up, two SurePath samples were taken from these women; in the laboratory, they were pooled before the testing, which was undertaken on samples diluted 1:1 in SurePath. Women with abnormal cytology or a positive HC2 test result at follow-up in 18 months (corresponding to the routine in the laboratory at the time of the study) were recommended for colposcopy. The last follow-up sample was received in the laboratory in June 2013. Follow-up was ascertained by linkage to the national Patobank in December 2013, allowing all women with abnormal repeated tests at least six months to attend for colposcopy. Hence, all histology was ascertained in approximately 2.5 years from the baseline testing, which, for women below age 50 years, corresponded to an almost entire screening interval. CIN lesions detected in HPV-positive women with follow-up other than study samples were included in the analysis. All colposcopies were undertaken by private or hospital-based gynecologists following routine protocols recommending biopsies from all suspicious areas, and random biopsies from the four quadrants if lesions were not visible.

### Cytology

Routine cytological evaluation of SurePath samples was undertaken first by FocalPoint Slide Profiler (BD, Burlington, NC). Blinded to the outcomes of HPV testing, samples were thereafter evaluated by cytoscreeners using FocalPoint GS Imaging System (BD), and abnormal findings were adjudicated by pathologists. Cytology was reported using the Bethesda 2001 system.

### HPV testing

All assay testing was undertaken in strict accordance with the protocols agreed upon with all manufacturers prior to the study, as described in detail previously [3–5,8,9]. HC2 testing was undertaken on the post-quot LBC material; cobas, CLART, and APTIMA testing was undertaken on the original (diluted) residual material. HC2 detects, collectively, the 13 HPV genotypes defined as high-risk by the International Agency for Research on Cancer [11]. The assay is based on hybridisation of HPV DNA to a high-risk HPV RNA probe cocktail. No re-test range was used. Cobas is a real-time PCR analysis detecting the 13 high-risk HPV genotypes and HPV66. The assay separately identifies HPV16 and HPV18, while the remaining 12 genotypes are detected collectively. CLART is a PCR-based low density array assay detecting 35 defined genotypes including the 13 high-risk genotypes. All genotypes are reported individually. APTIMA detects E6/E7 mRNA expression of the 13 high-risk HPV types and HPV66 collectively.

### Ethics statement

Baseline testing on the residual material was undertaken as a quality development study. In Denmark, such studies do not require ethical approval. Invitation to follow-up of HPV-positive/cytology-normal women was approved by the Ethical Committee of the Danish Capital Region (H-4-2012-120). It was not permitted to reveal the results of the baseline testing in the invitation letter, but women could obtain them from their general practitioner (GP), where they signed their consent forms. Women could also obtain their HPV test result without study participation. The study was notified to the Danish Data Inspection Agency (notification number 2010-41-5594).

### Statistical analysis

A positive HPV test was defined according to the manufacturers’ recommendations (HC2: relative light unit per cut off value ≥1; cobas channels 16, 18, and other high risk genotypes: cycle threshold values ≤40.5, ≤40.0, and ≤40.0, respectively; APTIMA: signal to cut off value ≥0.5). CLART was considered positive if at least one of the 13 high-risk HPV genotypes was detected. Abnormal cytology was defined as ≥ASCUS.

For each of the five screening tests, we determined the proportion of women with high-grade CIN (detected at any point during the study) having a positive test result at baseline. A positive screening test result indicates an increased risk of CIN lesions, but CIN is detectable only in a proportion of all screen-positive women. Hence, a false-positive screening test result was defined with respect to the histological outcome, i.e. as a positive test result at baseline that was not followed by a diagnosis of high-grade CIN. The number of women attending a colposcopy was determined as the number of women with a positive screening test result having at least one biopsy regardless of its diagnosis. The number of women with only repeated testing was determined as the number of women with a positive screening test result who had only cytology or HPV testing in their follow-up. We calculated the relative proportions of women with positive screening test results, with a colposcopy, with only repeated testing, with low-grade and high-grade CIN detected, and with false-positive test results for each screening test by comparing them to those of the reference screening test (cytology). To compare the screening tests to one another, two-sided p-values were calculated using McNemar test for discordant pairs. Persistent infection was defined as a (pooled) high-risk positive HPV test result at baseline and at follow-up on the same assay. We calculated the positive predictive value (PPV) of a persistent HPV infection as the proportion of women with a persistent infection having high-grade CIN. Positive test results on cobas were additionally analyzed by channel; in case of multiple positive channels, the results were reported hierarchically for the most “severe” channel (channel 16 > channel 18 > channel other high-risk). The effect of loss to follow-up on the detection of ≥CIN2 and ≥CIN3 was determined by assuming that the detection would have been the same in women without follow-up as it was observed in women who were followed up. For this calculation, cytology results were grouped as normal/ ASCUS/ LSIL/ ASC-H, AGC, HSIL, AIS.

## Results

### Study population at baseline

**Fig 1:**
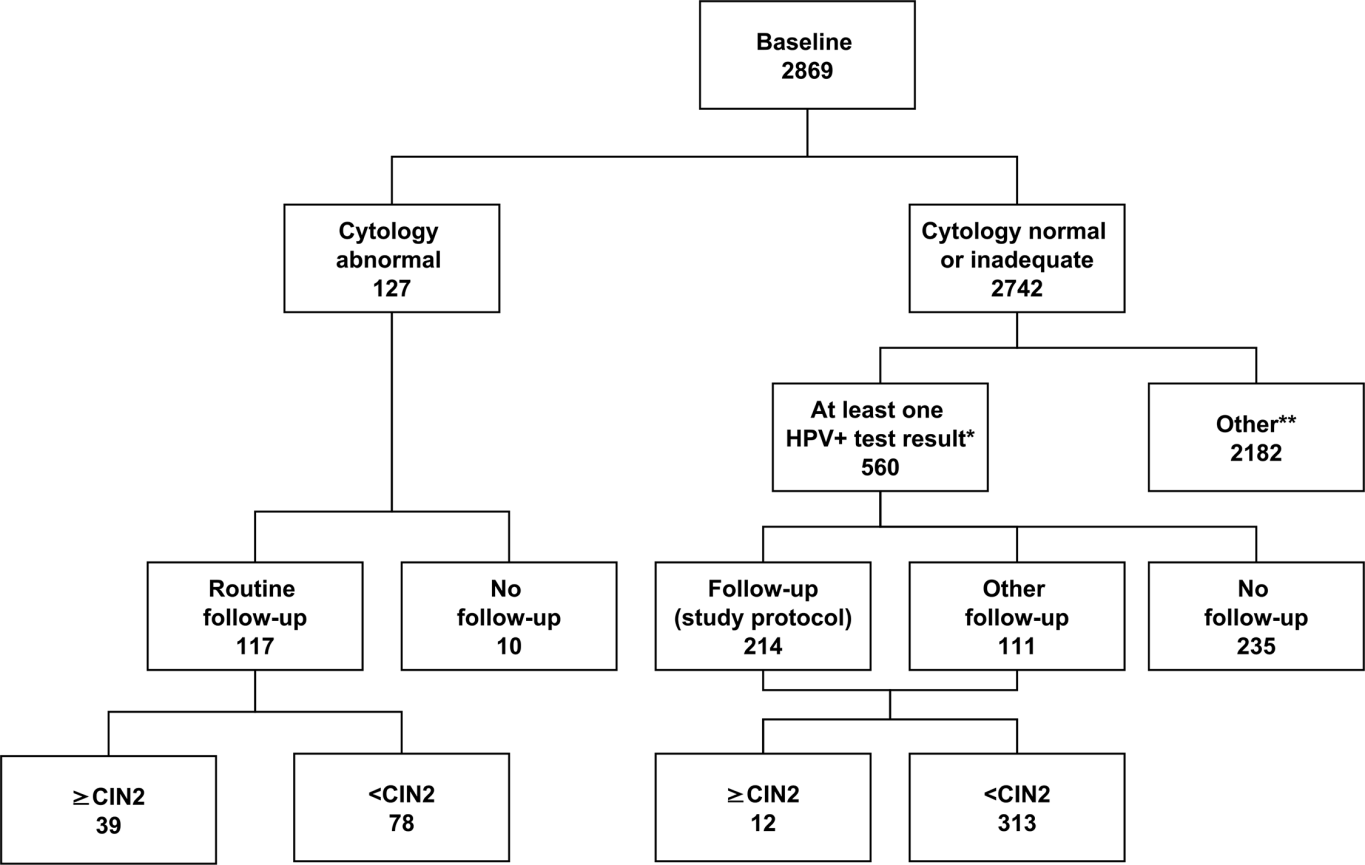
Description of the study. * Women with normal cytology. ** Other: HPV negative women on all four HPV assays and women with inadequate cytology. There were 13 women with inadequate cytology, of whom one was lost to follow-up, nine had normal follow-up cytology, and three had abnormal follow-up cytology without further testing; all had negative test results on all four HPV assays.

Of the 5,034 women included in the Horizon study, 2,869 (57.0%) were screened at 30–65 years (Table 1, Fig 1), with a mean age of 42.7 years. The remaining 2165 women were screened at a different age, or were undergoing follow-up for recent cervical abnormalities. In total, 560 (19.5%) women had normal cytology with a positive test result on at least one of the four HPV assays, and 127 (4.4%) had abnormal cytology. Samples of 336 (11.7%) women tested positive on HC2, either with normal or abnormal cytology. This was 465 (16.2%) for cobas, 453 (15.8%) for CLART, and 271 (9.4%) for APTIMA.

**Table 1:** Description of the 2,869 women aged 30–65 years with screening samples at baseline.

Characteristics at baseline	N (%)
Total	2,869 (100%)
**Age (years)**	
30–39	1,295 (45.1%)
40–49	897 (31.3%)
50–59	462 (16.1%)
60–65	215 (7.5%)
**Cytological diagnosis**	
Inadequate	13 (0.5%)
Normal	2,729 (95.1%)
ASCUS	63 (2.2%)
LSIL	32 (1.1%)
AGC, ASC-H, AIS, HSIL, or carcinoma	32 (1.1%)
**HPV test results**	
Positive on HC2	336 (11.7%)
Positive on cobas	465 (16.2%)
Positive on CLART	453 (15.8%)
Positive on APTIMA	271 (9.4%)
**Combined cytology and HPV results**	
Cytology normal and all 4 HPV test results negative	2,161 (75.3%)
Cytology normal and at least one HPV test result positive^a^	560 (19.5%)
Cytology abnormal, regardless of HPV test results	127 (4.4%)
Other^b^	21 (0.7%)

Abbreviations: AGC = atypical glandular cells, AIS = adenocarcinoma in situ, ASCUS = atypical squamous cells of undetermined significance, ASC-H = atypical squamous cells cannot exclude HSIL, HC2 = Hybrid Capture 2, HPV = Human Papillomavirus, HSIL = high-grade squamous intraepithelial lesions, LSIL = low-grade squamous intraepithelial lesions.

^a^ Invited for repeated testing in approximately 1.5 year according to the study protocol.

^b^ Inadequate cytology, or normal cytology with at least one invalid HPV test result and no positive HPV test result.

### Completeness of follow-up

Of the 127 women with abnormal baseline cytology, 117 (92%) were followed up (Table 2). Among 560 women with normal baseline cytology and a positive test result on at least one of the four HPV assays, this was 325 (58%). Of the latter 325 women, 214 (38% of all cytology-normal/HPV-positive women) had repeated testing as per study protocol (Table 3), and 111 (20%) women had other follow-up. Women with normal baseline cytology and a positive HPV test result who had follow-up were of a similar mean age (mean = 40.4, SD = 8.7) as women without follow-up (mean = 39.7, SD = 9.2). Completeness of follow-up was similar for all HPV assays. Reasons for lack of follow-up were known for 23 cytology-normal/HPV-positive women: 10 emigrated, nine were pregnant, two were living at an unknown address, one had a protected personal identification number, and one died.

**Table 2:** Follow-up outcomes for women with positive screening test results at baseline.

Baseline screening test result		Total N at baseline (column %)	Worst outcome during follow-up (row %)								
Cytology	HPV test		No follow-up	No histology		Histology					
				Normal cytology and/or negative HPV testing	Abnormal cytology and/or positive HPV testing	Inadequate histology	No CIN (CIN 0)	CIN1^a^	CIN2	CIN3	Cervical cancer
Normal or abnormal (total)	Negative or positive (total)	2,869 (100%)	1,863 (65%)	669 (23%)	39 (1%)	27 (1%)	183 (6%)	37 (1%)	12 (<1%)	38 (1%)	1 (<1%)
Normal	At least one of the four positive	560 (20%)	235 (42%)	217 (39%)	16 (3%)	9 (2%)	51 (9%)	20 (4%)	4 (1%)	8 (1%)	0 (0%)
**Cytology**											
Abnormal	Negative or positive	127 (4%)	10 (8%)	32 (25%)	4 (3%)	4 (3%)	23 (18%)	15 (12%)	8 (6%)	30 (24%)	1 (1%)
**HC2**											
Normal or abnormal	Positive	336 (12%)	103 (31%)	102 (30%)	12 (4%)	9 (3%)	41 (12%)	23 (7%)	11 (3%)	34 (10%)	1 (<1%)
Normal	Positive	250 (9%)	97 (39%)	90 (36%)	12 (5%)	6 (2%)	26 (10%)	10 (4%)	4^b^ (2%)	5 (2%)	0 (0%)
Abnormal	Positive	86 (3%)	6 (7%)	12 (14%)	0 (0%)	3 (3%)	15 (17%)	13 (15%)	7 (8%)	29 (34%)	1 (1%)
**Cobas**											
Normal or abnormal	Positive	465 (16%)	164 (35%)	157 (34%)	15 (3%)	8 (2%)	43 (9%)	29 (6%)	12 (3%)	37 (8%)	0 (0%)
Normal	Positive	386 (13%)	159 (41%)	145 (38%)	13 (3%)	6 (2%)	32 (8%)	19 (5%)	4^b^ (1%)	8^b^ (2%)	0 (0%)
Abnormal	Positive	79 (3%)	5 (6%)	12 (15%)	2 (3%)	2 (3%)	11 (14%)	10 (13%)	8 (10%)	29 (37%)	0 (0%)
**CLART**											
Normal or abnormal	Positive	453 (16%)	156 (34%)	152 (34%)	14 (3%)	8 (2%)	48 (11%)	26 (6%)	12 (3%)	36 (8%)	1 (<1%)
Normal	Positive	377 (13%)	152 (40%)	142 (38%)	13 (3%)	5 (1%)	38 (10%)	15 (4%)	4^b^ (1%)	8^b^ (2%)	0 (0%)
Abnormal	Positive	76 (3%)	4 (5%)	10 (13%)	1 (1%)	3 (4%)	10 (13%)	11 (14%)	8 (11%)	28 (37%)	1 (1%)
**APTIMA**											
Normal or abnormal	Positive	271 (9%)	95 (35%)	69 (25%)	9 (3%)	7 (3%)	28 (10%)	17 (6%)	12 (4%)	33 (12%)	1 (<1%)
Normal	Positive	200 (7%)	90 (45%)	61 (31%)	9 (5%)	5 (3%)	19 (10%)	7 (4%)	4^b^ (2%)	5^b^ (3%)	0 (0%)
Abnormal	Positive	71 (2%)	5 (7%)	8 (11%)	0 (0%)	2 (3%)	9 (13%)	10 (14%)	8 (11%)	28 (39%)	1 (1%)

Abbreviations: CIN = cervical intraepithelial neoplasia, HC2 = Hybrid Capture 2, HPV = Human Papillomavirus.

Note: Had the follow-up of women with abnormal screening tests been 100% complete, more ≥CIN2 would have been detected. The estimated numbers are as follows. For ≥CIN2: 41.3 (cytology), 53.5 (HC2), 59.3 (cobas), 58.8 (CLART), 55.4 (APTIMA). For ≥CIN3: 32.6 (cytology), 39.5 (HC2), 43.9 (cobas), 43.6 (CLART), 39.5 (APTIMA).

^a^ Includes atypia on histology and CIN not otherwise specified.

^b^ One case was diagnosed in women with follow-up outside of the study protocol, i.e. in women with cytology-normal/HPV-positive test results at baseline who did not have repeated testing in 18 months (as per the Horizon study’s protocol), but had additional testing for other (unknown) reasons.

**Table 3:** Women with normal cytology and at least one positive HPV test result at baseline: 1.5-year follow-up, persistence of infection, and final histology after repeated testing as per study protocol, by screening test.

Screening test	Cytology normal and HPV positive at baseline		Women with persistent infections/ incident abnormal cytology		
	Total	With 1.5-year follow-up (%)	N (% of those with follow-up)	≥CIN2 (% of those with persistent infection)	≥CIN3 (% of those with persistent infection)
Cytology	560^a^	214 (38%)	18 (8%)^b^	6 (33%)	4 (22%)
HC2	250	97 (39%)	46 (47%)	7 (15%)	4 (9%)
cobas	386	140 (36%)	68 (49%)^c^	≥9 (13%)^d^	≥6 (9%)^d^
CLART	377	147 (39%)	52 (35%)^c^	≥9 (17%)^d^	≥6 (12%)^d^
APTIMA	200	65 (33%)	24 (37%)	≥6 (25%)^d^	≥3 (13%)^d^

Abbreviations: CIN = cervical intraepithelial neoplasia, HC2 = Hybrid Capture 2, HPV = Human Papillomavirus.

^a^ Positive test result on any of the four HPV assays.

^b^ Two women had inadequate cytology.

^c^ 65/68 (96%) of women with a persistent infection on cobas had HPV genotypes detected on the same channel; 46/52 (88%) of women with a persistent infection on CLART had at least one high-risk genotype detected both at baseline and at 18-month follow-up.

^d^ Only women with abnormal cytology and/or a positive HC2 test result were referred for colposcopy.

### Persistence of HPV infections

The 214 women with cytology-normal/HPV-positive samples at baseline had repeated testing in on average 18.1 months (SD = 1.5, range: 15–22). Less than half of the initially HPV-positive women had persistent infections at repeated testing, i.e. a positive (pooled) high-risk HPV test result followed by another (pooled) high-risk positive HPV test result in 1.5 year, using the same assay. This varied between 35% (52/147) for CLART and 49% (68/140) for cobas. Most women with persistent infections had the same genotypes detected at baseline and at follow-up in 18 months. On cobas, 96% (65/68) of women with persistent infections had genotypes detected on the same channel, and on CLART, 88% (46/52) of women with persistent infections had at least one high-risk genotype detected both at baseline and at follow-up testing. The PPV for ≥CIN2 of persistent HPV infections was between 13 and 25% for all four assays; for ≥CIN3, this was between 9 and 13%. Of the same 214 women (all with normal cytology at baseline), 18 (8%) had incident cytological abnormalities at follow-up. The PPV of abnormal incident cytology were 33% and 22%, respectively.

### Detection of high-grade CIN by HPV assays

Of the 39 ≥CIN3, HC2 detected 35 (90%; 1.2% of all screened women), cobas and CLART each detected 37 (95%; 1.3% of all screened women), APTIMA detected 34 (87%, 1.2% of all screened women, Table 2). These differences were not statistically significant (McNemar p values >0.05). For comparison, cytology detected 31 (79%, 1.1% of all screened women). On cobas, 21 (57%) out of 37 ≥CIN3 were detected on the channel with other high-risk genotypes, 12 (32%) on channel 16, and the remaining 4 (11%) on channel 18 (S1 Table).

The one case of cervical cancer was detected by HC2, CLART, and APTIMA (and cytology), but not by cobas. Cobas returned a negative test result for this woman, with a CT value of the internal control of 28.9 indicating sufficiency of the sample for a valid analysis. In this woman, CLART detected an infection with HPV16.

For all four assays, normal or CIN1 histology at colposcopy was observed predominantly among HPV-positive/cytology-normal women. The proportion of false-positive test results for ≥CIN3 varied between 8.3% for APTIMA and 14.9% for cobas. The differences between the assays were significant, with a higher proportion of false-positive test results for cobas and CLART, and lower for APTIMA, compared to HC2 (McNemar p values all <0.001).

When HPV assays were compared to one another using ≥CIN2 as the endpoint, similar patterns were observed, with no statistically significant differences in the detection and a statistically significant gradient in the proportion of false-positive test results.

### HPV assays and cytology as primary screening tests

Using the outcomes from the Horizon study, we can compare the performance of routine cytology-based screening combined with HC2 triage of ASCUS, and primary HPV-based screening combined with cytology triage. With cytology screening, 127 (4.4%) women were recommended for further follow-up (Table 4). With HPV testing, this number ranged between 271 (9.4%) with APTIMA and 465 (16.2%) with cobas, i.e. approximately 2–4 times higher than with cytology, depending on the assay. In total, 2.8% of women were referred for colposcopy following cytology-based screening. With APTIMA, this was 3.4%, i.e. about 20% more than with cytology; this difference was significant. With the three DNA assays, the increases in the referral to colposcopy of at least 50% compared to cytology were also statistically significant. Furthermore, 1.3% of women screened with cytology had only repeated testing after abnormal cytology–but with HPV assays combined with cytological triage, this was twice as high with APTIMA and almost five times as high with cobas.

**Table 4:** Comparison of HPV assays with cytology among 2869 women with screening samples at age 30–65 years.

	Positive test results	Follow-up procedures^a^		Detection of CIN			False-positive test results	
		Colposcopy^b^	Repeated testing only	CIN1^c^	≥CIN2	≥CIN3	Endpoint: ≥CIN2	Endpoint: ≥CIN3
**Absolute numbers, N (%)**								
Cytology	127 (4.4%)	81 (2.8%)	36 (1.3%)	15 (0.5%)	39 (1.4%)	31 (1.1%)	88 (3.1%)	96 (3.3%)
HC2	336 (11.7%)	119 (4.1%)	114 (4.0%)	23 (0.8%)	46 (1.6%)	35 (1.2%)	290 (10.1%)	301 (10.5%)
cobas	465 (16.2%)	129 (4.5%)	172 (6.0%)	29 (1.0%)	49 (1.7%)	37 (1.3%)	416 (14.5%)	428 (14.9%)
CLART	453 (15.8%)	131 (4.6%)	166 (5.8%)	26 (0.9%)	49 (1.7%)	37 (1.3%)	404 (14.1%)	416 (14.5%)
APTIMA	271 (9.4%)	98 (3.4%)	78 (2.7%)	17 (0.6%)	46 (1.6%)	34 (1.2%)	225 (7.8%)	237 (8.3%)
**Relative proportion compared to cytology**								
HC2	2.7	1.5	3.2	1.53	1.18	1.13	3.3	3.1
cobas	3.7	1.6	4.8	1.93	1.26	1.19	4.7	4.5
CLART	3.6	1.6	4.6	1.73	1.26	1.19	4.6	4.3
APTIMA	2.1	1.2	2.2	1.13	1.18	1.10	2.6	2.5
**Discordant results between HPV assays and cytology: HPV+/cytology-, HPV-/cytology+**^**d**^								
HC2	(250, 41)***	(51, 13)***	(102, 24)***	(10, 2)*	(9, 2)	(5, 1)	(241, 39)***	(245, 40)***
cobas	(386, 48)***	(69, 21)***	(158, 22)***	(19, 5)**	(12, 2)*	(8, 2)	(374, 46)***	(378, 46)***
CLART	(377, 51)***	(70, 20)***	(155, 25)***	(15, 4)*	(12, 2)*	(8, 2)	(365, 49)***	(369, 49)***
APTIMA	(200, 56)***	(40, 23)*	(70, 28)***	(7, 5)	(9, 2)	(5, 2)	(191, 54)***	(195, 54)***

Abbreviations: CI = confidence interval, CIN = cervical intraepithelial neoplasia, HC2 = Hybrid Capture 2.

^a^ The difference between the total number of women with positive screening test results and women with either colposcopy or repeated testing only were women who had no follow-up.

^b^ Measured as registered with a biopsy in the Patobank.

^c^ Includes CIN1, histological atypia and CIN NOS.

^d^ *** P <0.001, ** P<0.01, * P<0.05.

In total, ≤1.0% of screened women had CIN1 detected by any of the five screening tests, and this was the lowest with cytology and APTIMA. The three HPV DNA assays led to a statistically significant increase compared to cytology. The detection of ≥CIN3 was between 10% and 19% higher (not statistically significant), and that of ≥CIN2 between 18% and 26% higher for the four HPV assays compared to cytology. The detection of ≥CIN2 was statistically significantly higher for cobas and CLART than it was for cytology, with the differences for HC2 and APTIMA approaching significance. With just over 1% of women having ≥CIN3 detected, 3.3% of all women screened with cytology had a false-positive test result. Using any of the four HPV assays, the proportions of women with false-positive test results were statistically significantly higher, and ranged from more than doubling with APTIMA to more than quadrupling with cobas.

Had the follow-up of abnormal test results been complete for all five screening tests, the differences between cytology and HPV assays in the number of women with a colposcopy referral and repeated testing would have increased. The differences in the detection of high-grade CIN between the HPV assays would have remained relatively small, within a range of ca. 11% higher detection estimated for cobas vs. APTIMA and HC2. The differences between the HPV assays and cytology would have become more visible; for example, the largest observed relative proportion of women with detected ≥CIN3, being 1.19 for cobas vs. cytology, would have increased to 1.35 (data not tabulated).

On cobas alone, approximately two-thirds of the colposcopy referrals (64%), repeated testing (66%) and false-positive test results (68%) were due to the results on the “other high-risk” channel alone (data not tabulated).

## Discussion

### General findings

In Danish women undergoing primary cervical screening at 30–65 years, the detection of high-grade CIN was similar for HC2, cobas, CLART, and APTIMA. With the total follow-up of about 2.5 years and additional testing of cytology-normal/HPV-positive women, these four HPV assays detected more high-grade CIN than cytology. Owing to small numbers, however, the differences were not always significant. HPV assays combined with cytological triage led to more referrals for colposcopy and biopsies with <CIN2, particularly in women with normal cytology. All four HPV assays referred more women to repeated testing compared to the current standard of cytology, and led to significantly more false-positive screening test results.

### Strengths and weaknesses of the study

To our knowledge, Horizon was the only study set in routine practice that compared more than two HPV assays in the same women while also ascertaining the histological status of women with cytology-normal/HPV-positive test results. Most previous studies compared two HPV assays at a time using ThinPrep samples, whereas this was the first multi-assay study using the other widely used LBC medium, SurePath. Unless women moved out of Denmark, all histology could be accounted for by the linkage to the nationwide Patobank. Histology was obtained and read under routine circumstances. Baseline samples were representative for the routine screening population [9]. They were tested while they were fresh [3,4], thereby further strengthening the applicability of the results for future routine HPV-based screening.

At the time of the baseline study, testing protocols for the handling of SurePath samples were only available for HC2, whereas the processing protocols for cobas, CLART, and APTIMA were agreed upon with the manufacturers. The manufacturers have since changed certain aspects of these protocols to support the testing also on SurePath samples, reflecting accumulation of experience with the assays’ performance on this cytology medium. In concordance with the protocol and by approval from the manufacturers, we diluted the original samples approximately 1:1 to obtain sufficient material for split sample testing by CLART, cobas and APTIMA. This can be seen as a weakness of the study, however, all three assays rely on testing aliquots of 0.5 to 1 ml out of the typically 10–20 ml available from liquid based cytology media or microbiology sampling liquids. Hence, the robustness of the assays should clearly be able to handle sampling variability in terms of cellularity.

Study size was defined by the laboratory’s practical considerations, and in agreement with the manufacturers. The highest observed detection rate of ≥CIN3 for any of the evaluated HPV assays was 37/2869 (1.3/100), and the lowest was for cytology, 31/2869 (1.1/100). An ex-post power calculation for this difference, taking into account the paired study design, revealed that approximately twice as many women would need to be included in the study to detect this difference at 80% power and α = 0.05. To detect the maximal difference in the detection rate between HPV assays alone, 37/2869 (1.3/100) vs. 34/2869 (1.2/100), the study size would need to be more than four times as large.

Of the women with cytology-normal/HPV-positive test results at baseline, 58% had follow-up. Very similar proportions of follow-up completeness were observed in randomized controlled trials comparing HPV testing with cytology in primary cervical screening [12]. Had the follow-up been complete (in the Horizon study and in the trials), more high-grade CIN lesions would have been detected in cytology-normal women, increasing the sensitivity in favour of HPV testing. Nevertheless, the gap between the numbers of women with high-grade CIN and positive test results in our study suggests that the high proportions of false-positive test results would not considerably decrease even with full follow-up.

Cytology normal women with positive HPV test results were recommended for repeated testing in 18 months. Although the repeated testing was, as the baseline testing, undertaken with all five screening tests, the women were at this stage managed according to cytology and HC2 alone. This strategy may have missed high-grade CIN in women with normal cytology and a negative HC2 test result but a persistent infection on the remaining three HPV assays; their number was, most likely, modest.

### Comparison with other studies

A number of studies have reported a direct comparison of cobas or APTIMA to HC2 or cytology. However, most often these studies were from referral populations where the observed differences between HPV assays were much smaller than in primary screening [9], thus emphasising the value of study designs such as that from the Horizon. The evidence from the randomized controlled trials (using HC2 or GP5+/6+ PCR assays) showed that HPV testing offers better protection from cervical cancer to screened women than cytology [1], but at the cost of substantially more false-positive tests and low-grade CIN diagnoses even in women aged ≥30 years [13]. Other screening studies also showed an increased detection of high-grade CIN using either HC2 [14,15], cobas [14,16], or APTIMA [17–19] compared to cytology. The differences in the detection of CIN between the three assays were small [14,17–21], yet the frequency of false-positive cobas [16] and HC2 [22] test results was two to three times higher compared with cytology. For APTIMA [18,19], the increases in false-positive test results compared to cytology were more variable and were smaller in settings with less specific cytology.

Horizon was the first study to evaluate CLART in primary screening. In previous studies including referral populations [23,24], this assay showed mixed results in terms of the sensitivity for high-grade CIN. In our study, CLART showed the same sensitivity as the three compared HPV assays. This is not surprising given that CLART, like e.g. cobas, utilizes modified HPV L1 consensus primers for target amplification. However, inter-study differences in DNA extraction methodology should also be taken into account. CLART, unlike e.g. cobas, relies on DNA extraction supplied by a third party.

### Clinical implications

In the Horizon study, the difference in the detection of high-grade CIN between HPV assays and cytology was not substantial (although to some extent this observation may have been due to the study size and an incomplete follow-up of women with abnormal screening tests). In Denmark, the quality of routine cytology may be relatively high. In our laboratory, cytopathology technicians undergo continued education by biennially participating in anonymous testing of adjudicated cytology samples. Upon reading the routine slides, the women’s complete screening histories as registered in the Patobank are available to cytopathology technicians as background information. The laboratory has defined standard operating procedures for cytology sample inadequacy. However, cases of cervical cancer with recent normal cytology are not uncommon in Denmark [25,26]. Moreover, in the large Dutch POBASCAM trial, the difference in the detection of ≥CIN3 was also not significant, relative detection for the intervention vs. control arm 1.15 (95% CI: 0.92–1.43) [27]. Nevertheless, the resulting rate of interval cancers was still significantly lower, 0.36 (95% CI: 0.14–0.91) [1]. In POBASCAM, the detection of CIN2 alone was significantly increased, 1.48 (95% CI: 1.09–2.04) for the intervention vs. control arm [27]. Also in our study, HPV testing detected about 50% more CIN2 (Table 2). It is, therefore, likely that also in Denmark HPV screening may turn out to provide better protection from cervical cancer than cytology.

The expectedly better protection from cervical cancer will come at a cost. Our data, supported with data from the randomized trials [13,28] and other studies [29], observed more false-positive test results with HPV-based than with cytology-based screening. Despite cytology triage, referral for colposcopy was more frequent (particularly for the DNA assays), and so was repeated testing. HPV testing led to more <CIN2 biopsies. Overdiagnosis of CIN2 and CIN3 with HPV screening has also been suggested [30]. Hence, although HPV testing is certainly beneficial in terms of preventing cervical cancer in screened women, there is still work to do for the currently available HPV assays in order to fine-tune HPV-based screening and improve the balance between the gains and the side effects. One of the strategies will be to optimize the criteria for a referral to colposcopy, e.g. by utilizing HPV genotyping, a change in the test thresholds, cytology, or other biomarkers. To decrease the overall frequency of false-positive test results, however, it will be particularly useful to find biomarkers that will allow to safely disregard a large proportion of positive HPV test results without increasing the risk of cervical cancer. To disregard a proportion of abnormal screening tests has been accepted by the scientific and clinical communities before–most recently, the evidence became convincing enough to ignore ASCUS diagnoses in case of a concurrent negative HPV test result.

Owing to the restrictions imposed by the Ethical Committee approval, women with normal cytology and positive HPV tests at baseline could only obtain the information on their HPV status through their GP. In routine HPV-based screening, women would be informed directly about their HPV status, so the loss to follow-up would probably be lower than observed in our study. Adequate follow-up of abnormal screening tests is one of the cornerstones of cervical cancer screening. There may be several reasons why this follow-up is often incomplete. One is that women might not be adequately informed about the abnormality. To this end, Danish women will from 2016 onwards receive their screening test results by email into the nationally mandated “e-box”. Another reason for the loss to follow-up might be that the abnormalities are not perceived as serious by the women themselves, or by their doctors. As an example, only 44% of Dutch women with borderline abnormal cytology were followed up on time when the definition of this diagnosis was broad and included inflammation and dysplastic changes [31]. When the definition was changed to include only the more risky dysplastic changes, the proportion with on-time follow-up almost doubled, possibly because the diagnosis was now perceived as more serious than before. If it is correct that HPV infections without cytological abnormalities might be considered as less critical than cytological abnormalities are at present, more effort should be made to provide adequate information on the nature of the abnormalities and the associated risks.

In conclusion, differences in the detection of high-grade CIN, false-positive test results, and the number of follow-up procedures were observed between HPV assays (HC2, cobas, CLART, and APTIMA) on the one hand, and cytology on the other hand in Danish women undergoing primary screening at age 30–65 years. The differences between cytology and HPV assays would have probably been larger had the follow-up of HPV-positive women been complete. The differences were small between the HPV assays alone, although HC2 and particularly APTIMA were associated with smaller increases in false-positive tests, number of needed colposcopies, and frequency of repeated testing than cobas and CLART.

## Supporting Information

S1 Table Breakdown of testing results by cobas channel. (DOCX)
