# Supplementary material for: Human Papillomavirus Assays and Cytology in Primary Cervical Screening of Women Aged 30 Years and Above
Source: PLoS One. 2016 Jan 20;11(1):e0147326. doi: 10.1371/journal.pone.0147326 (PMC4720421; doi:10.1371/journal.pone.0147326)
Supplement: S1 Table — (DOCX) [file pone.0147326.s001.docx]

27 October 2015

**Human Papillomavirus Assays and Cytology in Primary Cervical Screening of Women Aged 30 Years and Above**

**Short title: HPV Assays for Cervical Screening at ≥30 Years**

Matejka Rebolj,^1,*,#^ Jesper Bonde,^2,3^ Sarah Preisler,^2,3^

Ditte Ejegod,^2^ Carsten Rygaard,^2^ Elsebeth Lynge^1^

^1^ Department of Public Health, University of Copenhagen, Copenhagen, Denmark

^2^ Department of Pathology, Copenhagen University Hospital, Hvidovre, Denmark

^3^ Clinical Research Centre, Copenhagen University Hospital, Hvidovre, Denmark

^#^ Current address: Clinical Research Centre, Copenhagen University Hospital, Hvidovre, Denmark

*Corresponding author, e-mail: [matejka.rebolj@regionh.dk](mailto:matejka.rebolj@regionh.dk) (MR)

**Supporting information**

**S1 Table. Breakdown of testing results by cobas channel.**

**Appendix S1**

S1 Table. Breakdown of testing results by cobas channel.

| **Baseline screening test result** | | **Total N at baseline (column %)** | **Worst outcome during follow-up (row %)** | | | | | | | | |
| --- | --- | --- | --- | --- | --- | --- | --- | --- | --- | --- | --- |
| **Cytology** | **HPV test** |  | **No follow-up** | **No histology** | | **Histology** | | | | | |
|  |  |  |  | **Normal cytology and/or negative HPV testing** | **Abnormal cytology and/or positive HPV testing** | **Inadequate histology** | **No CIN (CIN 0)** | **CIN1^a^** | **CIN2** | **CIN3** | **Cervical cancer** |
| **Channel 16** |  |  |  |  |  |  |  |  |  |  |  |
| Normal or abnormal | Positive | 104 (4%) | 31 (30%) | 35 (34%) | 4 (4%) | 2 (2%) | 9 (9%) | 8 (8%) | 3 (3%) | 12 (12%) | 0 (0%) |
| Normal | Positive | 87 (3%) | 30 (34%) | 33 (38%) | 4 (5%) | 2 (2%) | 8 (9%) | 5 (6%) | 1 (1%) | 4 (5%) | 0 (0%) |
| Abnormal | Positive | 17 (1%) | 1 (6%) | 2 (12%) | 0 (0%) | 0 (0%) | 1 (6%) | 3 (18%) | 2 (12%) | 8 (47%) | 0 (0%) |
| **Channel 18** |  |  |  |  |  |  |  |  |  |  |  |
| Normal or abnormal | Positive | 50 (2%) | 17 (34%) | 18 (36%) | 2 (4%) | 1 (2%) | 5 (10%) | 2 (4%) | 1 (2%) | 4 (8%) | 0 (0%) |
| Normal | Positive | 43 (1%) | 17 (40%) | 18 (42%) | 2 (5%) | 1 (2%) | 4 (9%) | 0 (0%) | 0 (0%) | 1 (2%) | 0 (0%) |
| Abnormal | Positive | 7 (0%) | 0 (0%) | 0 (0%) | 0 (0%) | 0 (0%) | 1 (14%) | 2 (29%) | 1 (14%) | 3 (43%) | 0 (0%) |
| **Channel other high-risk** |  |  |  |  |  |  |  |  |  |  |  |
| Normal or abnormal | Positive | 311 (11%) | 116 (37%) | 104 (33%) | 9 (3%) | 5 (2%) | 29 (9%) | 19 (6%) | 8 (3%) | 21 (7%) | 0 (0%) |
| Normal | Positive | 256 (9%) | 112 (44%) | 94 (37%) | 7 (3%) | 3 (1%) | 20 (8%) | 14 (5%) | 3 (1%) | 3 (1%) | 0 (0%) |
| Abnormal | Positive | 55 (2%) | 4 (7%) | 10 (18%) | 2 (4%) | 2 (4%) | 9 (16%) | 5 (9%) | 5 (9%) | 18 (33%) | 0 (0%) |

Abbreviations: CIN=cervical intraepithelial neoplasia, HPV=Human Papillomavirus.
